# Genetic variant in a BaP-activated super-enhancer increases prostate cancer risk by promoting AhR-mediated *FAM227A* expression

**DOI:** 10.7555/JBR.37.20230049

**Published:** 2024-02-27

**Authors:** Lulu Fan, Hao Wang, Shuai Ben, Yifei Cheng, Silu Chen, Zhutao Ding, Lingyan Zhao, Shuwei Li, Meilin Wang, Gong Cheng

**Affiliations:** 1 Department of Environmental Genomics, Jiangsu Key Laboratory of Cancer Biomarkers, Prevention and Treatment, Collaborative Innovation Center for Cancer Personalized Medicine, School of Public Health, Nanjing Medical University, Nanjing, Jiangsu 211166, China; 2 Department of Genetic Toxicology, the Key Laboratory of Modern Toxicology of Ministry of Education, Center for Global Health, School of Public Health, Nanjing Medical University, Nanjing, Jiangsu 211166, China; 3 Department of Urology, the First Affiliated Hospital of Nanjing Medical University, Jiangsu Province Hospital, Nanjing, Jiangsu 210029, China

**Keywords:** super-enhancer, prostate cancer, genetic variants, AhR, BaP, *FAM227A*

## Abstract

Genetic variants in super-enhancers (SEs) are increasingly implicated as a disease risk-driving mechanism. Previous studies have reported an associations between benzo[a]pyrene (BaP) exposure and some malignant tumor risk. Currently, it is unclear whether BaP is involved in the effect of genetic variants in SEs on prostate cancer risk, nor the associated intrinsic molecular mechanisms. In the current study, by using logistic regression analysis, we found that rs5750581T>C in 22q-SE was significantly associated with prostate cancer risk (odds ratio = 1.26, *P* = 7.61 × 10^−5^). We also have found that the rs6001092T>G, in a high linkage disequilibrium with rs5750581T>C (*r*^2^ = 0.98), is located in a regulatory aryl hydrocarbon receptor (AhR) motif and may interact with the *FAM227A* promoter in further bioinformatics analysis. We then performed a series of functional and BaP acute exposure experiments to assess biological function of the genetic variant and the target gene. Biologically, the rs6001092-G allele strengthened the transcription factor binding affinity to AhR, thereby upregulating *FAM227A*, especially upon exposure to BaP, which induced the malignant phenotypes of prostate cancer. The current study highlights that AhR acts as an environmental sensor of BaP and is involved in the SE-mediated prostate cancer risk, which may provide new insights into the etiology of prostate cancer associated with the inherited SE variants under environmental carcinogen stressors.

## Introduction

Prostate cancer ranks the second most common cancer and the fifth leading cause of cancer-related deaths in males worldwide^[1]^. Patients with prostate cancer can be effectively treated with androgen deprivation therapy. Unfortunately, nearly all patients eventually develop resistance to the above-mentioned therapy and progress to castration-resistant prostate cancer (CRPC)^[2]^. The development and progression of prostate cancer stem from combined effects of genetic factors, lifestyle, and environmental factors^[3]^, among which genetic factors play an important role. Genome-wide association studies (GWAS) have identified nearly 270 known loci associated with risk of prostate cancer^[4]^. However, there are many single nucleotide polymorphisms (SNPs) found by GWAS in noncoding regions with unknown functions, and 64% of these SNPs are found in enhancer regions^[5]^.

Enhancers are a class of *cis*-acting noncoding DNA regulatory elements that regulate promoter activity by the binding of specific transcription factors^[6]^. Super-enhancers (SEs) are clusters of enhancers that span tens of kilobases of the genome^[5]^. Compared with typical enhancers, SEs have a higher enrichment of transcription factors and epigenetic markers, and drive stronger transcriptional activation^[7]^. SEs exert vital regulatory roles in biological processes, such as carcinogenesis, cell differentiation, and immune responses, including those in prostate cancer^[8]^. In addition, studies have demonstrated that disease-risk SNPs are enriched in SE regions, explicitly influencing cancer risk and associated pathogenic processes by interfering with the binding of transcription factors and distal regulation of target genes^[9]^. However, few studies have focused on associations between genetic variants in SEs and prostate cancer risk or CRPC progression.

Transcription factor binding to the enhancers is a critical step in transcriptional activation^[10]^. Environmental factors influence environmentally sensitive transcription factors (*e.g.*, the aryl hydrocarbon receptor, AhR; the estrogen receptor, ER) and therefore influence tumor development^[11–12]^. AhR is a multifunctional regulatory protein that senses and responds to polycyclic aromatic hydrocarbons (*e.g.*, benzo[a]pyrene, BaP) and persistent planar halogenated polycyclic hydrocarbons (*e.g.*, 2,3,7,8-tetrachlorodibenzo-p-dioxin, TCDD) as well as a ligand-activated transcription factor that facilitates tumor progression and disease tolerance^[13]^. It is believed that ligand activation increases the nuclear translocation of AhR and its binding with a xenobiotic response element associated with the aryl hydrocarbon receptor nuclear translocator to modulate gene expression^[14]^. BaP is recognized as an important environmental carcinogen, which induces several human cancers, including cancers of the colorectum and breast^[15–16]^. One study indicated that the effects of BaP in prostate cancer might be mainly due to its AhR-mediated activity rather than the activation of the DNA damage response^[17]^. Nevertheless, the epigenetic mechanism of AhR and BaP in prostate cancer remains unclear.

In the current study, we used a comprehensive strategy combining bioinformatics analysis and laboratory experiments to investigate the association between genetic variants in SEs and prostate cancer risk as well as its epigenetic mechanism under BaP exposure.

## Subjects and methods

### Study population

The current study included 4662 prostate cancer cases and 3114 control subjects from the Prostate, Lung, Colorectal and Ovarian (PLCO) Cancer Screen Trial. Detailed information on the PLCO Trial has been presented previously^[18]^. The baseline information on the cases and controls is provided in ***Supplementary Table 1*** (available online). The procedures were approved and carried out following the ethical standards of the National Cancer Institute and the local institutional review board as well as the Helsinki Declaration.

### Detection of SEs

Histone H3 lysine 27 acetylation (H3K27ac) is a commonly used marker of SEs. Thus, the H3K27ac chromatin immunoprecipitation sequencing (ChIP-seq) data was used to identify SEs. We downloaded H3K27ac ChIP-seq data of two prostate cancer cell lines LNCaP (GSM1902615), PC-3 (GSE96399), and normal prostate tissue (GSE143079) from the Gene Expression Omnibus (GEO) database. Then, we aligned the sequencing reads to long reference sequences by Bowtie2 and performed quality control by SAMtools. The Model-based Analysis for ChIP-Seq (MACS2) with the default settings was applied to call peaks. Next, the rank-ordering of super-enhancers (ROSE) algorithm was employed to identify SEs^[5]^. In brief, peak regions within 2.5 kb of annotated transcription start sites were removed, and the enhancers within a distance of 12.5 kb were further stitched. Finally, the enhancers were ranked by the H3K27ac signal level, and the data were plotted on a curve. The signal corresponding to the point of tangency on the tangent line with a slope of one is the threshold value for distinguishing typical enhancers from SEs; the enhancers with signals above the threshold value are SEs. After removing those identified in normal prostate tissue and LNCaP cells and those located on chromosome X, 767 SEs unique to PC-3 cells were retained for further analyses.

### Single nucleotide polymorphism genotyping

Illumina HumanHap300v1.1 and HumanHap250Sv1.0 (dbGaP accession: phs000207.v1.p1)^[19]^ and Illumina HumanOmni2.5 (dbGaP accession: phs000882.v1.p1)^[20]^ were used for DNA genotyping. Based on the 1000 Genome Project, IMPUTE 2 software was conducted for imputation.

### Selection of SNPs

**Figure 1:**
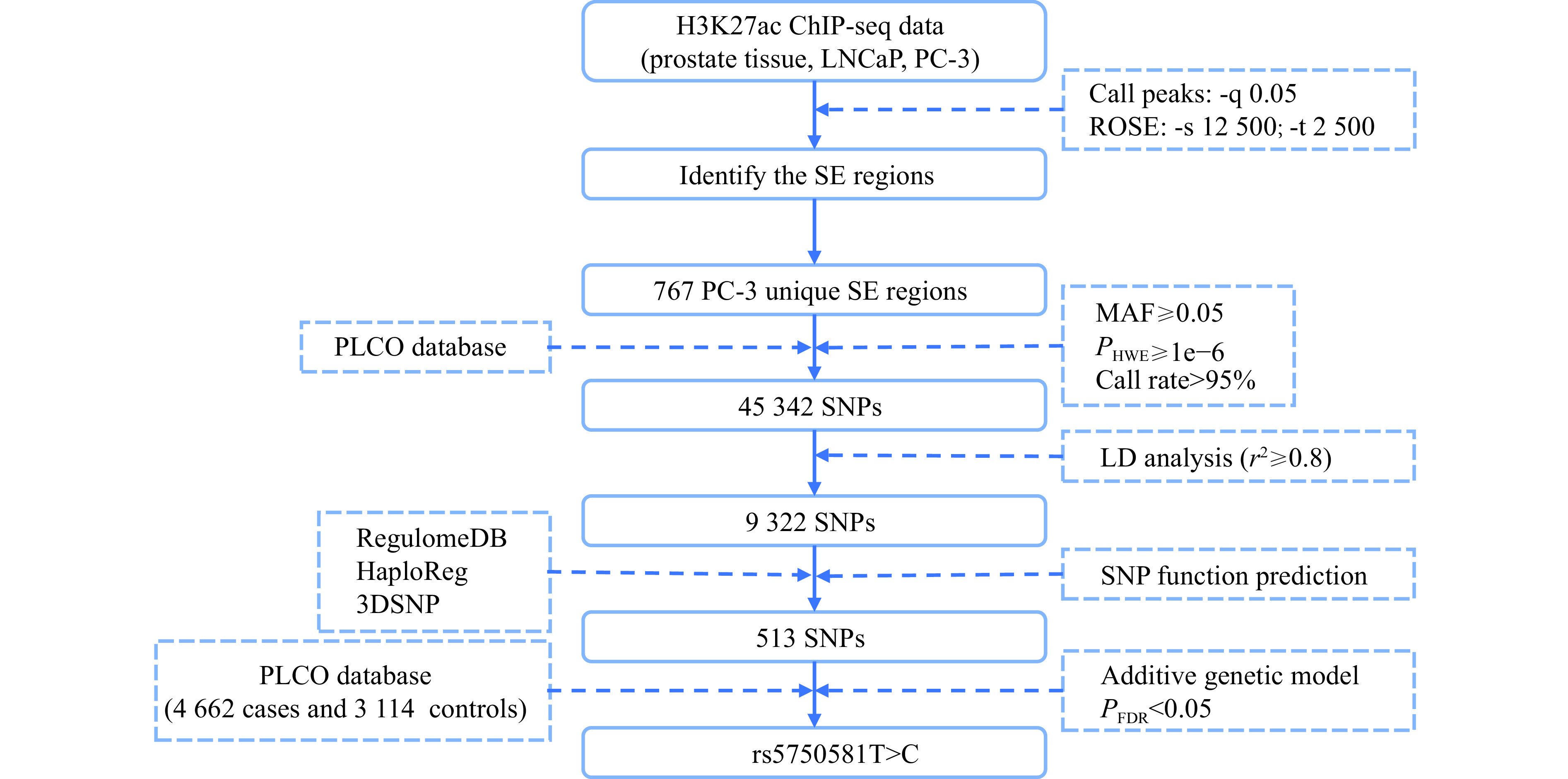
Flowchart for the selection of SNPs in SEs.

The process for screening candidate SNPs is shown in ***Fig. 1***. First, the 1000 Genomes Project and PLCO Trial were applied to select SNPs in SE regions that met the following inclusion criteria: (a) minor allele frequency (MAF) ≥ 0.05; (b) *P*-value of Hardy-Weinberg equilibrium (HWE) ≥ 1×10^−6^; and (c) call rate > 95%. After quality control, tag SNPs were selected using the linkage disequilibrium (LD) analysis (*r*^2^ ≥ 0.8). Then, we used RegulomeDB (http://regulomedb.org/), HaploReg (http://pubs.broadinstitute.org/mammals/haploreg/) and 3DSNP (http://omic.tech/3dsnpv2/) to predict the potential functions of SNPs. The scores of all selected SNPs in the RegulomeDB were less than 4. In HaploReg, the SNPs with selected expression quantitative trait locus (eQTL) hits, proteins bound, motifs, and the enrichment of enhancer histone marks or DNase hypersensitivity were verified. The transcription factor binding sites and enhancers of the selected SNPs were visualized with 3DSNP.

### Bioinformatics and gene expression analyses

The eQTL analysis was performed in the Genotype-Tissue Expression (GTEx) portal (http://www.gtexportal.org/) to select target genes regulated by susceptible SNPs. Gene expression data were downloaded from The Cancer Genome Atlas (TCGA) database (http://cancergenome.nih.gov/) and Gene Expression Omnibus (GEO) database (GSE200879, GSE70768) to calculate changes in the expression levels of the selected genes (log_2_ transformed). Histone modification on chromosomes was analyzed using the data from the University of California Santa Cruz (UCSC) Genome Browser (http://genome.ucsc.edu/).

### Cell lines and cell culture

The human prostate cancer cell lines LNCaP (androgen-dependent) and PC-3 (androgen-independent), along with the human normal prostatic epithelial cell line RWPE-1, were purchased from the Shanghai Institute of Biochemistry and Cell Biology, Chinese Academy of Sciences (Shanghai, China). LNCaP and PC-3 cells were cultured in RPMI-1640 medium and RWPE-1 cells were cultured in DMEM with supplemented 10% fetal bovine serum. All cells were maintained under 5% CO_2_ at 37 ℃.

### RNA interference and real-time reverse transcription-PCR (RT-qPCR)

siRNAs (GenePharma, Shanghai, China) were synthesized to knock down the expression of *FAM227A* and *AHR*, the sequences of which are listed in ***Supplementary Table 2*** (available online). As shown in ***Supplementary Fig. 1*** (available online), the siRNAs with the best interference efficiency (si*AHR*-1 and si*FAM227A*-3) were used in subsequent experiments.

Total RNAs from cells were harvested by RNA-easy isolation reagent (Vazyme, Nanjing, China) and measured by Nanodrop ND 2000 (Thermo Fisher Scientific, Waltham, MA, USA). Qualified RNAs were reverse transcribed to cDNA using Prime Script RT Master Mix (Takara, ShigaJ, Japan). AceQ qPCR SYBR Green Master Mix (Vazyme) was used to detect relative mRNA expression in a Roche Light Cycler480 Ⅱ system (Roche, Basel, Switzerland). The sequences of the primers are listed in ***Supplementary Table 3*** (available online), and *GAPDH* was used as an internal control.

### Cell proliferation and colony formation assays

LNCaP and PC-3 cell proliferation was observed with a Cell Counting Kit-8 (CCK-8; Dojindo, kumamoto, Japan) at scheduled time intervals. Utilizing an Infinite M200 spectrophotometer (Tecan, Männedorf, Switzerland), absorbance at 450 nm (OD450) was tested to quantify cell growth. In the colony formation assay, a density of 800 to 1000 cells was seeded into a 6-well plate and cultured for 10 to 14 days. Then, the colonies were fixed with 95% methanol and stained with 0.1% crystal violet (Beyotime, Shanghai, China).

### Transwell migration and invasion assays

For the migration assay, 3 × 10^4^ to 5 × 10^4^ cells were seeded in the upper chamber (Corning, Corning, NY, USA) and medium containing 10% FBS was added to the lower chamber of a Transwell. The cells were incubated for 48 h at 37 ℃ in 5% CO_2_. For the invasion assay, Matrigel (Yeasen, Shanghai, China) was layered in Transwell inserts and allowed to solidify at 37 ℃ for at least 4 h, and 6 × 10^5^ to 10 × 10^5^ cells were then seeded into the upper chamber to invade for 48 h. The subsequent procedures were the same as those used for colony formation assays.

### Flow cytometry

For analysis of cell cycle, cells were collected and immobilized with 70% ethanol at −20 ℃ for at least 18 h. Then, the cells were dyed with propidium iodide and sorted using an FACS Calibur flow cytometer (Beckman Coulter, Brea, CA, USA). For the apoptosis assay, cells were dyed with the components of an Annexin V-FITC Apoptosis Detection Kit (Invitrogen, Waltham, MA, USA), and the percentage of apoptotic cells was determined by flow cytometry.

### Dual-luciferase reporter assay

A 1000-bp fragment containing the rs6001092 T or G allele of the enhancer sequence (chr22: 38700594–38701594) and the *FAM227A* promoter region (chr22: 39052398–39053397) was synthesized and inserted into the pGL4.10 vector (Promega, Madison, WI, USA) at the *Nhe*Ⅰ and *Xho*Ⅰ restrictive sites. Relative luciferase activity was quantified as the ratio of firefly to Renilla luciferase activity.

### Electrophoretic mobility shift assay

Nuclear proteins were extracted from cells using NE-PER Nuclear and Cytoplasmic Extraction Reagents (Thermo Fisher Scientific). Then, nuclear extracts were incubated with synthetic 3′ biotin-labeled 23-bp oligonucleotides by using a LightShift Chemiluminescent Electrophoretic Mobility Shift Assay (EMSA) Kit (Thermo Fisher Scientific). The oligonucleotide sequences are shown in ***Supplementary Table 4*** (available online). The binding reactions were subjected to electrophoresis on a 6% polyacrylamide gel, and the products were detected by a chemiluminescent reaction with a stabilized streptavidin-horseradish peroxidase conjugate. Unlabelled probes were added to the reaction at a 200-fold excess for competition assays.

### Subcutaneous xenograft mouse models

Male nude mice (five to six weeks old) were subcutaneously injected in both flanks with PC-3 cells (5 × 10^6^) suspended in PBS. Tumor growth was examined every seven days. After five weeks, the mice were sacrificed, and the sizes and weights of tumors were measured. All the *in vivo* experiments were performed in accordance with institutional and national guidelines and approved by the Animal Ethical and Welfare Committee of Nanjing Medical University (IACUC-2212040).

### Cytotoxicity assay

LNCaP and PC-3 cells were treated with different concentrations of BaP (0 [DMSO control], 0.1, 1, 5, 10, 25, 50, or 100 μmol/L) for 24 h. At the indicated time point, serum-free medium and CCK8 reagent were mixed (10∶1), and the mixture (100 μL) was added to each well. The absorbance at 450 nm of each well was measured with an Infinite M200 spectrophotometer (Tecan).

### Immunofluorescence assay

Cells were seeded in a glass-bottom cell culture dish and treated with BaP or DMSO for 24 h. Then, the cells were fixed with 4% paraformaldehyde and permeabilized with 0.5% Triton X-100. After washing, the cells were blocked in 10% goat serum (Beyotime) at 37 ℃ for 1 h and then incubated with a rabbit anti-AhR (1∶50 dilution; Cat. #ET1703-11, HUABO, Hangzhou, China) at 4 ℃ overnight. Next, the cells were incubated with Alexa Fluor 647-conjugated anti-rabbit IgG (1∶500 dilution; Cat. #ab150075, Abcam, UK) at 37 ℃ for 2 h. Finally, nuclei were stained with DAPI (Beyotime). The cells were observed and captured using a confocal laser scanning microscope (Zeiss LSM700, Germany).

### Western blotting analysis

Cell protein were harvested using RIPA lysis buffer (Beyotime) supplemented with phenylmethanesulfonyl fluoride (Beyotime). The extracted proteins were separated by 10% SDS-polyacrylamide gel electrophoresis and transferred onto a polyvinylidene difluoride membrane (Millipore, St. Louis, MO, USA). The membranes were incubated with primary antibodies including anti-FAM227A (1∶1000 dilution; Cat. #STJ193640, St John's Laboratory, London, UK), anti-AhR (1∶1000 dilution; Cat. #ET1703-11, HUABIO), anti-CYP1A1 (1∶1000 dilution; Cat. #13241-1-AP, Proteintech, Wuhan, China), or anti-GAPDH (1∶1000 dilution; Cat. #AF0006, Beyotime) as the internal control. HRP-conjugated anti-rabbit or anti-mouse antibodies (1∶2000 dilution; Cat. #SA00001-1 or SA00001-2, Beyotime) were then used. Signals were observed and imaged by using an ECL system (Thermo Fisher Scientific).

### Statistical analysis

The differences in demographic characteristics between the cases and controls were evaluated by Student's *t*-test and the *χ*^2^ test. A logistic regression model was performed to evaluate associations between genetic variants and prostate cancer risk. Heterogeneity in stratification analyses was tested by Cochran's *Q* and *I*^2^ statistics. In addition, differences in mRNA expression were analyzed through the two-tailed Mann-Whitney *U* test or Student's *t*-test. All above analyses were performed with R 4.1.3 (http://r-project.org/) and PLINK 1.09 (https://www.cog-genomics.org/plink2/).

## Results

### Identification of prostate cancer-specific SEs

**Figure 2:**
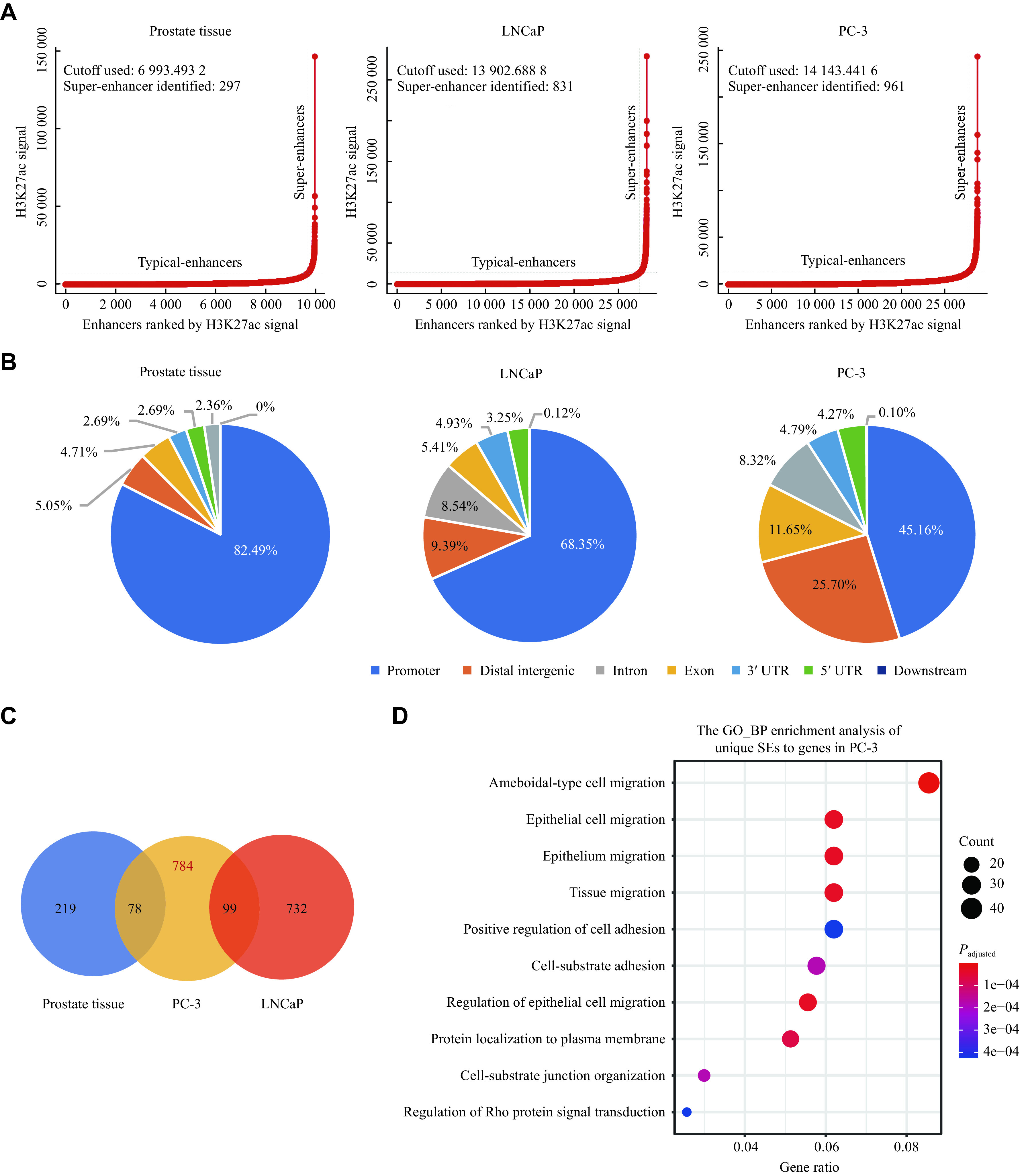
Identification of prostate cancer-specific SEs.

SEs in normal prostate tissue and cancer cells were identified through H3K27ac ChIP-seq. The ROSE algorithm identified 297, 831, and 961 SEs in normal tissue, LNCaP, and PC-3 cells, respectively (***Fig. 2A***). As expected, a considerable proportion of H3K27ac ChIP-seq peaks were located at distal intergenic and intronic regions (***Fig. 2B***). After the comparison among normal tissue and the two cell lines, we retained 784 PC-3-specific SE regions (***Fig. 2C***). In addition, Gene Ontology analysis showed that genes associated with the PC-3-specific SEs were enriched mainly in biological processes of migration (***Fig. 2D***), implying characteristics of the enhanced migration of PC-3 cells. After removing SEs located on chromosome X, 767 SEs were screened for further analyses.

### Associations between SNPs in cancer-specific SEs and prostate cancer risk

As indicated by the process shown in ***Fig. 1***, we comprehensively selected candidate SNPs in the SEs unique to PC-3 cells. A total of 45342 SNPs met the quality control criteria. Then, 513 SNPs remained after linkage disequilibrium (LD) analysis (*r*^2^ ≥ 0.8) and functional prediction. The retained SNPs were analyzed under an additive genetic model, and 24 SNPs were significantly associated with prostate cancer risk (*P*_adjusted_ < 0.05) (***Supplementary Table 5***, available online). After false discovery rate (FDR) correction, only rs5750581T>C was retained, with an odds ratio (OR) of 1.26 (*P*_FDR_ = 3.90 × 10^−2^).

The SE containing rs5750581 was found to be located in the 22q13.1 region, and we thus named this SE 22q-SE. In this region, three clumped SNPs of rs5750581 were identified by LD analysis (*r*^2^ ≥ 0.8) (***Supplementary Fig. 2A***, available online). Based on data for PC-3 cell line in the UCSC database, the regions of rs5750581 and the three clumped SNPs contained specific histone mark patterns, such as a high H3K27ac and histone H3 lysine 4 monomethylation (H3K4me1) enrichment and low histone H3 lysine 4 trimethylation (H3K4me3) enrichment, a DNase Ⅰ hypersensitivity, and transcription factors (***Supplementary Fig. 2B***, available online). Interestingly, RegulomeDB suggested that rs6001092T>G was located in the region of an AhR binding motif (***Supplementary Table 6*** and ***Supplementary Fig. 2C***, available online). Studies have indicated that AhR acts as an environmentally sensitive transcription factor to facilitate tumor progression^[12]^. Thus, after a comprehensive analysis of rs5750581 and its clumped SNPs based on functional characteristics, such as environmentally sensitive transcription factors, rs6001092 was speculated to biologically function as a tag SNP to affect prostate cancer risk.

### Stratification analysis of rs6001092 with prostate cancer risk

We performed an association analysis between rs6001092 and prostate cancer risk with four genetic models (codominant, additive, dominant, and recessive; ***Table 1***). The SNP rs6001092 was significantly associated with risk of prostate cancer in additive (OR = 1.25, 95% CI: 1.11–1.40, *P* = 1.89 × 10^−4^) and dominant genetic models (OR = 1.29, 95% CI: 1.13–1.46, *P* = 1.03 × 10^−4^). We then performed stratification analysis based on demographic and clinicopathologic characteristics with the dominant genetic model. As shown in ***Supplementary Table 7*** and ***Supplementary Fig. 3*** (available online), the G allele was associated with an increased prostate cancer risk in participants over 70 years old (OR = 1.26, 95% CI: 1.10–1.45, *P* = 8.82 × 10^−4^), ever or current smokers (OR = 1.33, 95% CI: 1.11–1.59, *P* = 1.87 × 10^−3^ for ever smokers; OR = 2.01, 95% CI: 1.31–3.10, *P* = 1.53 × 10^−3^ for current smokers), and participants without a family history of prostate cancer (OR = 1.29, 95% CI: 1.13–1.47, *P* = 1.75 × 10^−4^).

**Table 1:** Analyses of the association between rs6001092 and prostate cancer risk

Genotypes	Cases			Controls		OR (95% CI)	*P-*value	Adjusted OR (95% CI)^a^	*P*-value^a^
	*n*	%		*n*	%				
TT	3373	73.74		2334	76.42	1.00		1.00	
TG	1122	24.53		672	22.00	1.16 (1.04–1.29)	9.56×10^−3^	1.29 (1.13–1.47)	1.46×10^−4^
GG	79	1.73		48	1.58	1.14 (0.79–1.64)	4.82×10^−1^	1.27 (0.82–1.95)	2.78×10^−1^
Additive model						1.13 (1.03–1.25)	1.14×10^−2^	1.25 (1.11–1.40)	1.89×10^−4^
Dominant model						1.15 (1.04–1.28)	8.23×10^−3^	1.29 (1.13–1.46)	1.03×10^−4^
Recessive model						1.10 (0.77–1.58)	6.03×10^−1^	1.20 (0.78–1.84)	4.15×10^−1^
^a^Adjusted for age, body mass index, smoking status and family history of prostate cancer in logistic regression model. Abbreviations: OR, odds ratio; CI, confidence interval.									

The above-mentioned associations were further evaluated by the stratified subgroup analysis of clinicopathological characteristics (***Supplementary Table 8***, available online). In clinicopathologic subgroup stratification analyses, we observed that TG/GG was significantly associated with an enhanced prostate cancer risk in patients with a Gleason score of 7 (OR = 1.37, 95% CI: 1.16–1.63, *P* = 2.48 × 10^−4^), PSA 10–20 (OR = 1.53, 95% CI: 1.23–1.90, *P* = 1.53 × 10^−4^), and stage Ⅲ/Ⅳ (OR = 1.40, 95% CI: 1.10–1.78, *P* = 6.89 × 10^−3^) subgroups.

### The rs6001092 remotely modulated *FAM227A* expression by mediating the AhR-binding affinity

**Figure 3:**
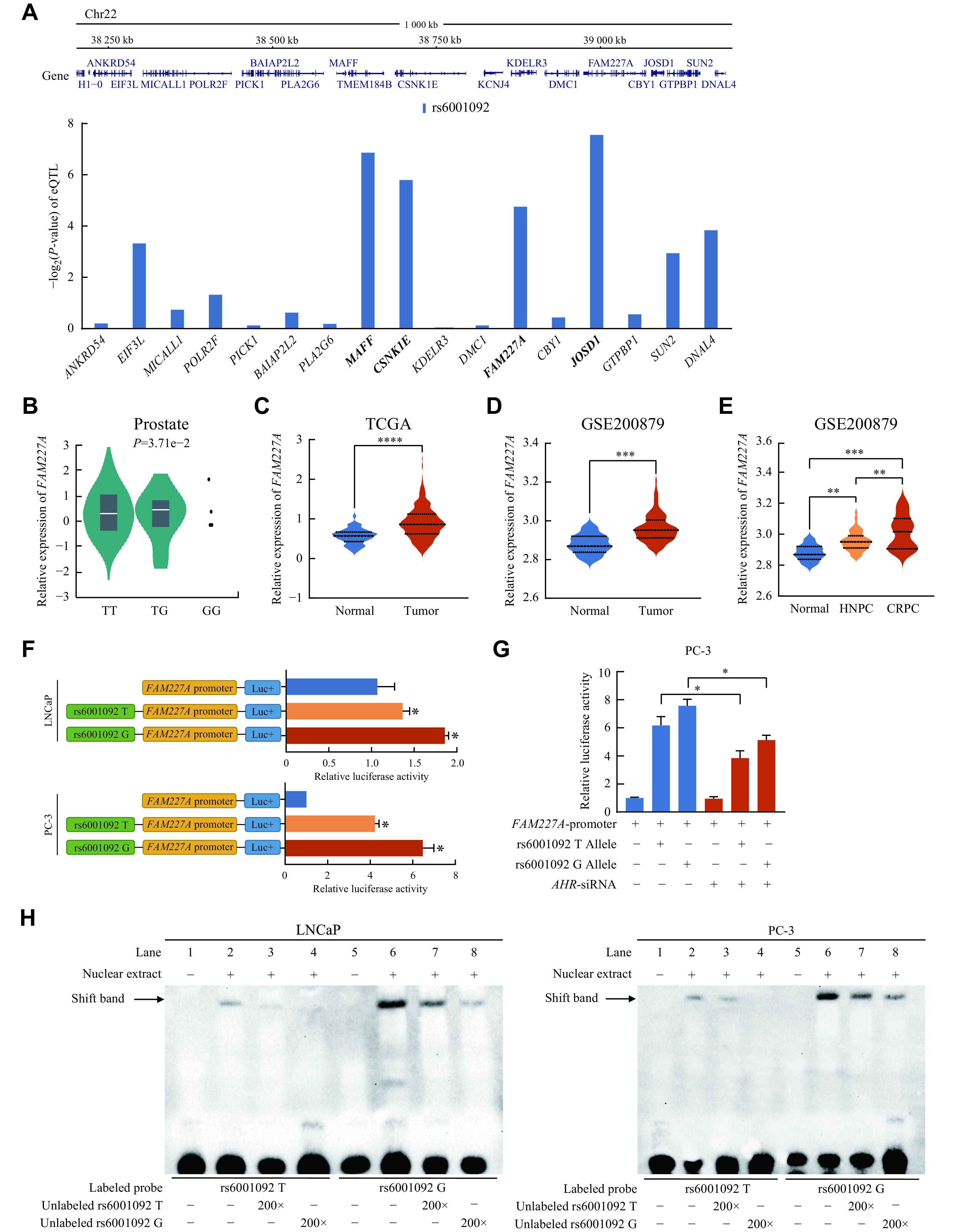
rs6001092 remotely modulated *FAM227A* expression by mediating the AhR binding affinity.

To evaluate genetic effects of rs6001092 on genes within the 1-Mb region, we conducted an eQTL analysis using the GTEx database. Significant associations were found between the rs6001092 genotype and the expression levels of four genes (*MAFF*, *CSNK1E*, *FAM227A*, and *JOSD1*; ***Fig. 3A***). According to the expression data of these four genes in TCGA, *CSNK1E* and *FAM227A* were differentially expressed in cancer tissues and normal tissues. Then, we sought to determine whether the direction of eQTL effects and tumor differences were consistent and found that only the expression of *FAM227A* was directionally concordant with the eQTL effect (***Fig. 3B*** and ***3C***, ***Supplementary Fig. 4*** [available online]). We further observed an increased expression of *FAM227A* in prostate cancer tissues, compared with normal tissues in the GSE200879 dataset (***Fig. 3D***). Notably, *FAM227A* expression was higher in CRPC tissues than in hormone-naive prostate cancer tissues in GSE200879 (***Fig. 3E***). Therefore, we hypothesized that *FAM227A* was a target gene regulated by rs6001092. As described above, rs6001092 was located in an intronic region enriched with histone enhancer marks (***Supplementary Fig. 2B***) and also located in a regulatory AhR motif (***Supplementary Fig. 2C***). Then, we predicted transcription factors in the rs6001092 region and the promoter region of *FAM227A* by JASPAR. Consistent with the above-mentioned findings, these results indicated that rs6001092 might remotely modulate *FAM227A* expression by altering the binding affinity of the transcription factor AhR to the enhancer region (***Supplementary Table 9***, available online).

We hypothesized that the enhancer covering rs6001092 regulated the transcription of *FAM227A* by altering the recruitment of AhR. Thus, we structured enhancer luciferase reporter vectors comprising the rs6001092 central region and the *FAM227A* promoter, respectively. The region containing rs6001092 showed a significantly stronger activation, as compared with the *FAM227A* promoter alone, suggesting that the region centered on rs6001092 acts as an enhancer of *FAM227A* (***Fig. 3F***). Consistent with the results of the eQTL analysis, the rs6001092 G allele showed a significantly higher enhancer activity than the T allele. Moreover, the knockdown of *AHR* reduced the transcriptional activity of *FAM227A* regulated by the rs6001092 T/G alleles, suggesting that rs6001092 may bind to the transcription factor AhR (***Fig. 3G***). In addition, an EMSA was also conducted to observe the differences in the binding affinities of the rs6001092 T and G alleles for AhR. These results also supported that the G allele had a stronger binding affinity than the T allele (***Fig. 3H***). Notably, the *AHR* expression was significantly higher in CRPC tissues than in hormone-naive prostate cancer tissues, or in cell lines (***Supplementary Fig. 5***, available online). Taken together, these results indicate that the elevated *AHR* expression in CRPC may be the reason for the high expression level of *FAM227A*; and SNP rs6001092 influences the transcriptional regulation of the AhR-mediated SE, thus altering the expression of *FAM227A*.

### *FAM227A* was upregulated and promoted malignant phenotypes of prostate cancer

Because the higher expression levels of *FAM227A* in prostate cancer tissues than in normal tissues in TCGA and the GSE200879 dataset was observed (***Fig. 3C*** and ***3D***), we then conducted stratification analyses based on clinical characteristics in the TCGA database to verify the association of *FAM227A* with the progression of prostate cancer. The expression of *FAM227A* was significantly higher (stratified by Gleason score, pathologic T stage, pathologic N stage, and biochemical recurrence status) in cancer subgroups than in normal tissue subgroup (*P*_all_ < 0.05; ***Supplementary Fig. 6***, available online). Moreover, the expression of *FAM227A* showed an increasing trend with an increasing Gleason score (***Supplementary Fig. 6A***); a higher pathologic T or N stage associated with a higher expression level of *FAM227A* (***Supplementary Fig. 6B*** and ***6C***); and the increased expression of *FAM227A* was significantly associated with biochemical recurrence (***Supplementary Fig. 6D***). Finally, to investigate potential molecules contributing to prostate cancer, we performed Gene Set Enrichment Analysis (GSEA) based on the differentially expressed genes between the *FAM227A* high- and low-expression cancer tissues. The signiﬁcantly enriched hallmark terms included MYC targets and cell cycle (***Supplementary Fig. 7***, available online).

**Figure 6:**
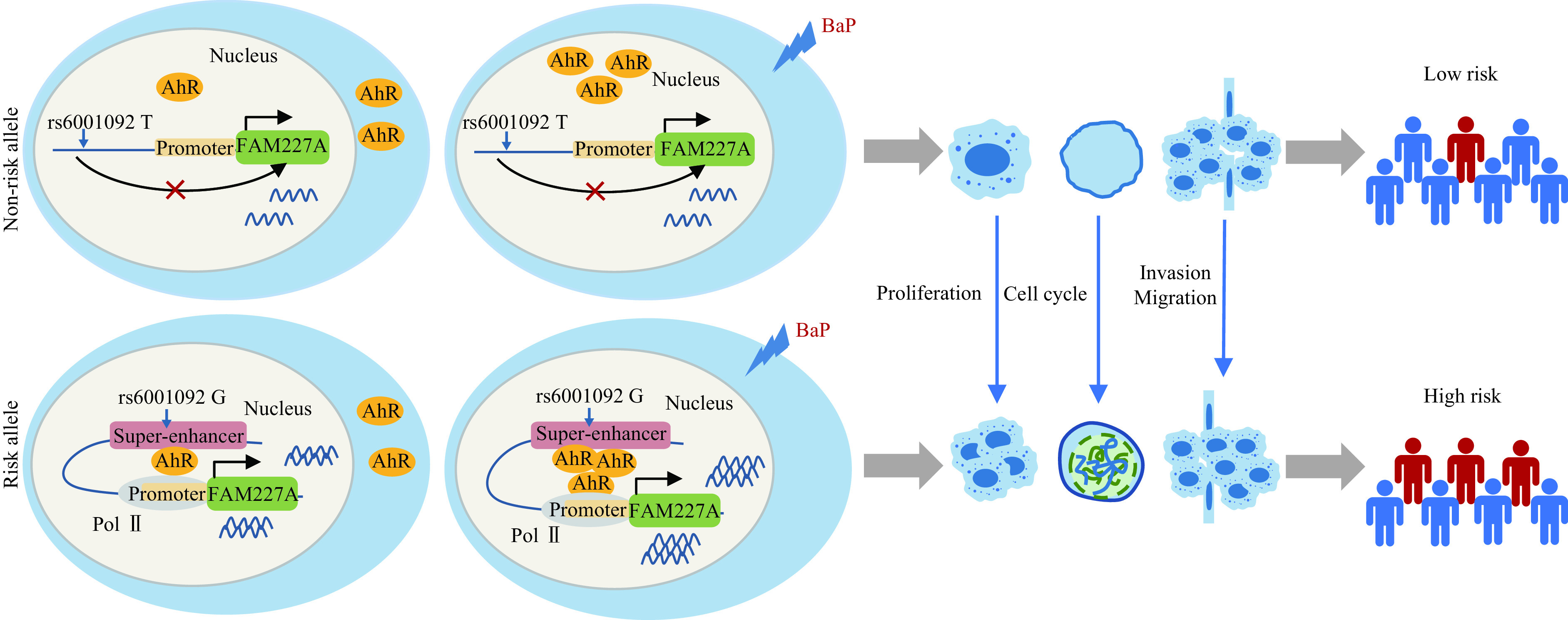
A proposed model of transcriptional regulation of 22q-SE in prostate cancer cells.

**Figure 4:**
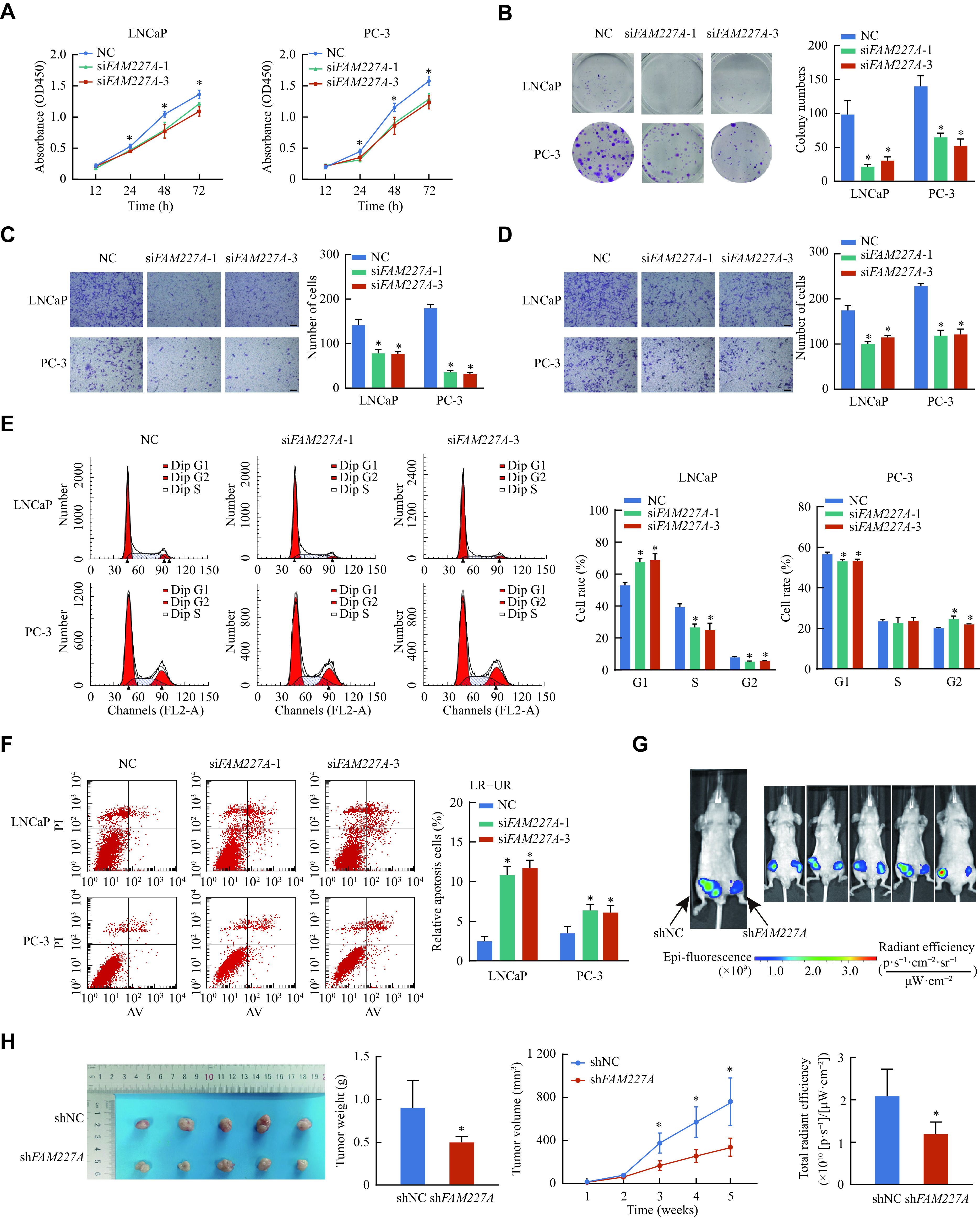
*FAM227A* is upregulated and promotes malignant phenotypes of prostate cancer cells.

To further explore the functions of *FAM227A* in prostate cancer, *FAM227A* siRNA (si*FAM227A*) was transiently transfected into LNCaP and PC-3 cells. Knockdown of *FAM227A* suppressed cell growth and decreased the colony formation ability (***Fig. 4A*** and ***4B***). Moreover, *FAM227A* deletion markedly reduced the invasion and migration abilities (***Fig. 4C*** and ***4D***). Additionally, flow cytometric analysis revealed that *FAM227A* deletion promoted apoptosis and affected the percentage of cells in the G1 and G2 phase (***Fig. 4E*** and ***4F***). Moreover, the xenograft tumor growth was remarkably inhibited in the sh*FAM227A* group, compared with the shNC group (***Fig. 4G*** and ***4H***).

### BaP promoted AhR-mediated SE activation and *FAM227A* expression

**Figure 5:**
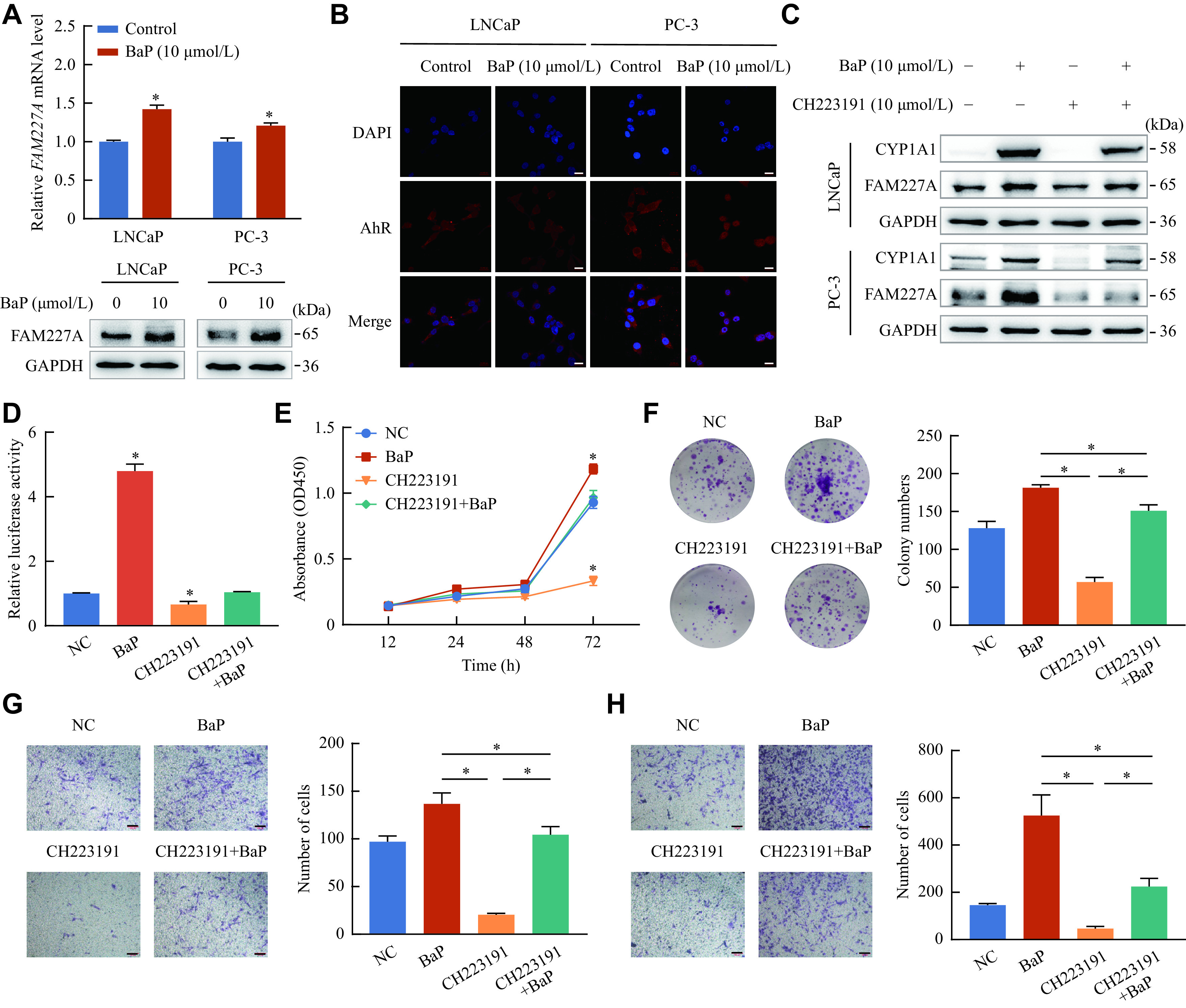
BaP promoted AhR-mediated SE activation and *FAM227A* expression.

To explore whether the activation of AhR increases the transcript level of *FAM227A* through 22q-SE, we treated LNCaP and PC-3 cells with BaP, an activator of AhR. First, the two cells were exposed to different concentrations of BaP, and the IC_20_ was calculated to be 10 μmol/L (***Supplementary Fig. 8A***, available online). According to the protein expression of CYP1A1, an indicator of AhR activation, 10 μmol/L BaP effectively activated AhR (***Supplementary Fig. 8B***, available online), and we thus selected 10 μmol/L BaP as the treatment concentration for further experiments. The mRNA and protein expression levels of FAM227A were significantly increased after BaP treatment (***Fig. 5A*** and ***Supplementary Fig. 9A*** [available online]). Immunofluorescence assays demonstrated that treatment with BaP facilitated AhR translocation to the nucleus (***Fig. 5B***).

To further verify whether BaP affects *FAM227A* expression and function through AhR, we used a specific AhR inhibitor, CH223191 (10 μmol/L), to block AhR activation in LNCaP and PC-3 cells. The Western blotting analysis revealed that the effect of BaP in promoting *FAM227A* expression was attenuated by CH223191 treatment in PC-3 cells, while CH223191 had a weak effect in LNCaP cells (***Fig. 5C*** and ***Supplementary Fig. 9B*** [available online]). In addition, a dual-luciferase reporter assay confirmed that *FAM227A* expression was significantly upregulated by BaP stimulation, while this tendency was also weakened by CH223191 treatment in PC-3 cells (***Fig. 5D***).

Further phenotypic experiments were performed by treating cells with BaP and CH223191 for 24 h. Compared with the negative control, BaP treatment promoted cell proliferation, colony formation, invasion, and migration. However, CH223191 treatment inhibited the effect of BaP treatment, suppressing cell proliferation, colony formation, invasion, and migration (***Fig. 5E***–***5H***). The flow cytometric analysis showed that the apoptosis of PC-3 cells was not significantly altered by either BaP or CH223191 treatment (***Supplementary Fig. 10A***, available online). Moreover, BaP or CH223191 treatment affected G2/M progression in PC-3 cells (***Supplementary Fig. 10B***, available online). In brief, after PC-3 cells were exposed to BaP, *FAM227A* expression and malignant biological behaviors were facilitated through the activation of AhR.

## Discussion

In the current study, through the bioinformatics analysis, we identified a prostate cancer risk-associated SNP, rs5750581, among the SEs specific to prostate cancer. We then found that rs6001092, in the high LD with rs5750581, interacted with the promoter of *FAM227A*. Biologically, the rs6001092-G allele altered the binding affinity of AhR, thereby upregulating *FAM227A* transcription. Further molecular biological experiments demonstrated that *FAM227A* affected the malignant phenotypes of prostate cancer. In addition, BaP induced these malignant behaviors by directly activating AhR (***Fig. 6***).

New avenues of studies on SEs have recently been opened in light of the rapid development of genome-wide sequencing technology. Recently, GWAS have shown that SEs are associated with many diseases. For example, rs539846 located in a SE affects chronic lymphocytic leukemia susceptibility through differential binding to RELA (NF-kappa-B subunit) and direct modulation of *BMF* expression, impacting the antiapoptotic protein B cell lymphoma 2 (BCL2)^[9]^. Moreover, many discoveries have been made regarding the effect of SEs on prostate cancer^[21–22]^. For instance, the acetylated homeobox B13 (HOXB13) plays a role in controlling *ACK1* gene transcription in prostate cancer by establishing a CRPC-specific SE, thereby affecting tumor growth^[22]^. In our current study, 22q-SE influenced prostate cancer susceptibility by altering the binding affinity of AhR and regulating the *FAM227A* expression.

Currently, the associations between genetic variants in SEs and prostate cancer risk have not been explored in depth. Here, we used a large-scale genetic association analysis in the PLCO trial database to identify genetic variants, revealing that rs5750581 and its clumped SNP rs6001092 were significantly associated with prostate cancer risk. SNP rs6001092 has specific histone marks and an environmentally sensitive transcription factor motif, indicating that rs6001092 may affect prostate cancer risk by exerting biological function of a tag SNP. In addition, one meta-analysis study showed that a history of smoking was implicated as a causative factor for prostate cancer, and the results in our stratification analysis were consistent^[23]^.

Abnormal transcription activities driven by SEs, such as cyclin dependent kinase 7 (CDK7), bromodomain and extra-terminal domain (BET)/bromodomain containing 4 (BRD4), and androgen receptor (AR), have been reported to influence prostate cancer phenotypes^[8]^. In the current study, AhR, as a ligand-activated transcription factor, was found to be involved in prostate carcinogenesis through genomic and epigenetic modifications^[13]^. AhR also binds to factors other than the aryl hydrocarbon receptor nuclear translocator, such as Kruppel-like factor 6 (KLF6), and identifies *cis* elements other than xenobiotic response elements, regulating the expression of multiple genes^[24]^. In functional experiments, the rs6001092-G allele was found to alter the binding affinity of AhR to upregulate *FAM227A* expression through the enhancer activity.

One genome scan study showed that the deletion region of chromosome 22q13, where *FAM227A* is located, might be associated with the risk of aggressive prostate cancer^[25]^. However, little is known about the biological roles of *FAM227A* in prostate tissues. The evidence provided by the current study indicates that *FAM227A* may be a prostate cancer inducer. Compared with that in normal tissues, the *FAM227A* expression was upregulated in prostate cancer tissues. Moreover, phenotypic experiments showed that *FAM227A* induced malignant behaviors in prostate cancer cells. In addition, GSEA showed that the MYC_targets and cell cycle-related gene sets (E2F_tragets and G2M_checkpoint) were activated, which were the well-known pathways of tumorigenesis and associated with metastatic behaviors^[26–27]^. For example, the MYC_targets promoted prostate tumorigenesis and progression by disrupting the release of transcriptional pausing of AR-regulated genes^[26]^. Thus, FAM227A might participate in the pathogenesis and development of prostate cancer by regulating these known pathways.

BaP acts as a canonical exogenous ligand for AhR^[28]^. Multi-target genes are transcriptionally regulated, when AhR translocates from the cytoplasm to the nucleus in response to the ligand activation^[29]^. One case-control study showed an elevated risk of high-grade prostate cancer to be associated with exposure to polycyclic aromatic hydrocarbons released from wood, including BaP^[30]^. Previous studies showed that BaP affected the JAK2/STAT3 pathway by activating AhR to promote prostate cancer progression^[31]^. In the current study, molecular assays indicated that the activation of AhR by BaP affected the regulation of *FAM227A* and malignant behaviors. Dietary intake is one of the major exposure pathways for BaP. For example, overcooked or smoked meat significantly increased prostate cancer risk^[32]^. In contrast, previous studies have observed a beneficial effect of a high adherence to the Mediterranean diet and the antioxidant role of olive oil on overall prostate cancer survival^[33–34]^. Therefore, improving dietary patterns may attenuate the effect of BaP on prostate cancer progression.

However, the current study has several limitations. First, a large-scale validation in other populations was needed to determine the effects of rs6001092. Second, we need biochemical experiments to investigate the mechanisms of SEs. Third, there is an insufficient number of population studies on the association between BaP exposure and prostate cancer risk.

In conclusion, our findings provide some evidence that rs6001092 in 22q-SE contributes to prostate cancer risk and development mediated by AhR and the upregulated *FAM227A* expression. Moreover, BaP affects the *FAM227A* expression by activating AhR and promoting enhancer activity, thereby inducing malignant behaviors of prostate cancer cells. The current study may provide a new strategy for preventing and screening prostate cancer by uncovering the etiology of prostate cancer associated with carcinogen stressors.

## SUPPLEMENTARY DATA

Supplementary data to this article can be found online.
