# Supplementary material for: Genetic variant in a BaP-activated super-enhancer increases prostate cancer risk by promoting AhR-mediated FAM227A expression
Source: J Biomed Res. 2024 Feb 27;38(2):149–62. doi: 10.7555/JBR.37.20230049 (PMC11001591; doi:10.7555/JBR.37.20230049)
Supplement: Supplementary file 1 — Supplementary data to this article can be found online. [file jbr-38-2-149-S1.pdf]

Available online at www.jbr-pub.org.cn

Open Access at PubMed Central

JBR

Journal of Biomedical Research, 2024 0(000): 1–10

Supplementary Data

Genetic variant in a BaP-activated super-enhancer increases prostate cancer risk by promoting AhR-mediated *FAM227A* expression

Lulu Fan[1,2,△], Hao Wang[1,2,△], Shuai Ben[1,2], Yifei Cheng[1,2], Silu Chen[1,2], Zhutao Ding[1,2], Lingyan Zhao[1,2], Shuwei Li[1,2], Meilin Wang[1,2,✉], Gong Cheng[3,✉]

[1]*Department of Environmental Genomics, Jiangsu Key Laboratory of Cancer Biomarkers, Prevention and Treatment, Collaborative Innovation Center for Cancer Personalized Medicine, School of Public Health, Nanjing Medical University, Nanjing, Jiangsu 211166, China;*
[2]*Department of Genetic Toxicology, the Key Laboratory of Modern Toxicology of Ministry of Education, Center for Global Health, School of Public Health, Nanjing Medical University, Nanjing, Jiangsu 211166, China;*
[3]*Department of Urology, the First Affiliated Hospital of Nanjing Medical University, Jiangsu Province Hospital, Nanjing, Jiangsu 210029, China.*

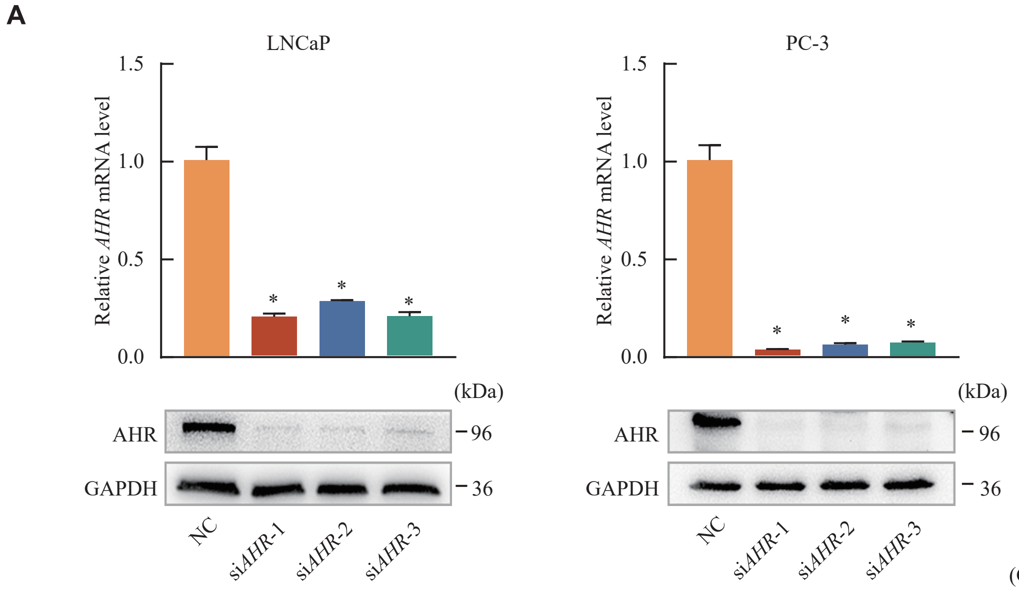

(Continued)

△These authors contributed equally to this work.

✉Corresponding authors: Gong Cheng, Department of Urology, the First Affiliated Hospital of Nanjing Medical University, 300 Guangzhou Road, Nanjing, Jiangsu 210029, China. E-mail: gcheng@njmu.edu.cn; Meilin Wang, Department of Environmental Genomics, School of Public Health, Nanjing Medical University, 101 Longmian Avenue, Jiangning District, Nanjing, Jiangsu 211166, China. E-mail: mwang@njmu.edu.cn.

Received: 06 March 2023; Revised: 29 May 2023; Accepted: 30 May 2023; Published online: 27 February 2024

CLC number: R737.25, Document code: A

The authors reported no conflict of interests.

This is an open access article under the Creative Commons Attribution (CC BY 4.0) license, which permits others to distribute, remix, adapt and build upon this work, for commercial use, provided the original work is properly cited.

© 2024 by Journal of Biomedical Research.

https://doi.org/10.7555/JBR.37.20230049

(Continued)

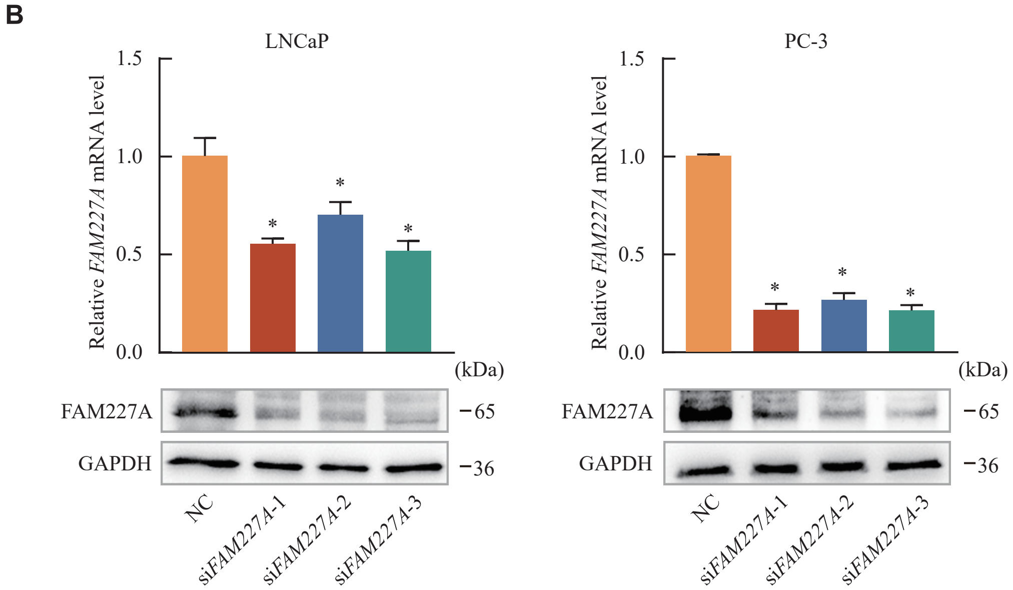

Supplementary Fig. 1 **Inhibition of siRNA on mRNA and protein expression levels of AHR and FAM227A.** A: si*AHR*-1 has the best interference efficiency and was selected for both PC-3 and LNCaP cells. B: si*FAM227A*-3 has the best interference efficiency and was selected for both LNCaP and PC-3 cells. Data are presented as mean ± standard deviation.$^*P$ < 0.05 compared with the control group by two-tailed Student's *t*-test.

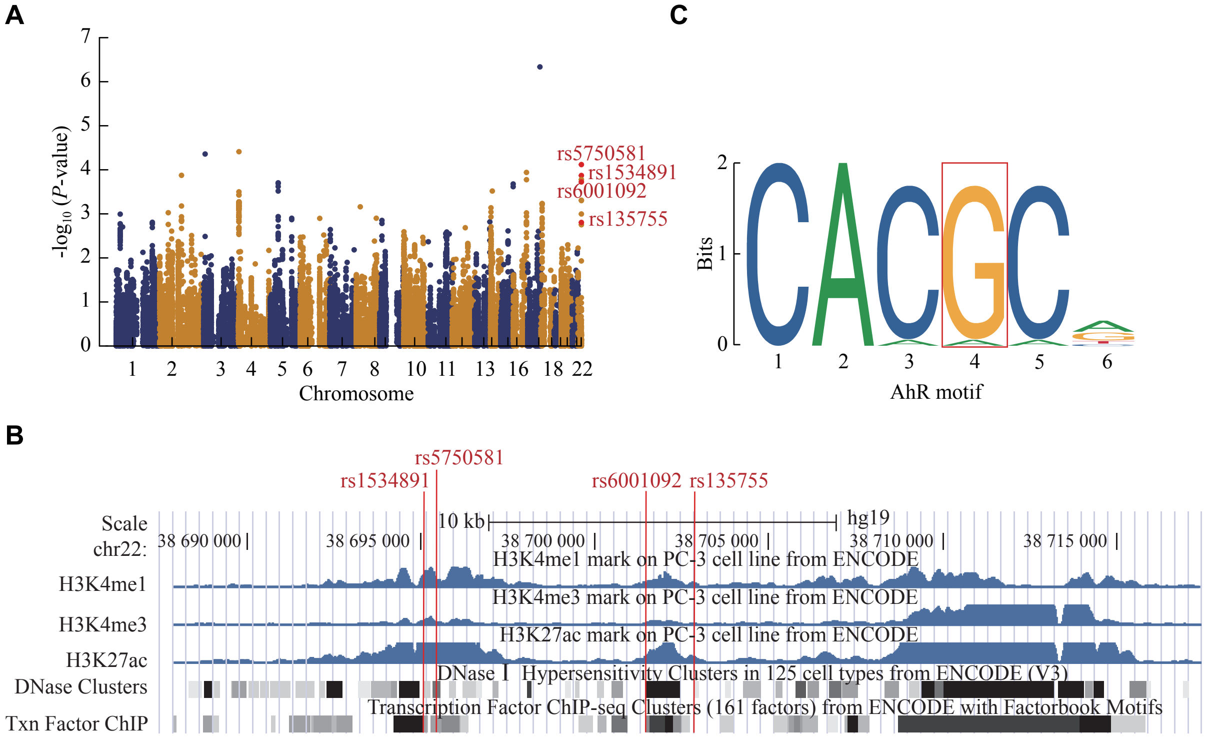

Supplementary Fig. 2 **Summary of *in silico* analyses for rs6001092.** A: Manhattan plot for association between SNPs in super-enhancers with prostate cancer risk. B: Overview of the epigenetic profiling of H3K4me1, H3K4me3, and H3K27ac chromatin modifications, distributions of DNase Ⅰ hypersensitivity clusters and transcription factor binding sites in the LD region for the human prostate cancer cell line PC-3 released by UCSC databases. C: AhR binding motif predicted through RegulomeDB. Abbreviations: SNP, single nucleotide polymorphism; H3K4me1, histone H3 lysine 4 monomethylation; H3K4me3, histone H3 lysine 4 trimethylation; H3K27ac, histone H3 lysine 27 acetylation; LD, linkage disequilibrium; ChIP-seq, chromatin immunoprecipitation sequencing.

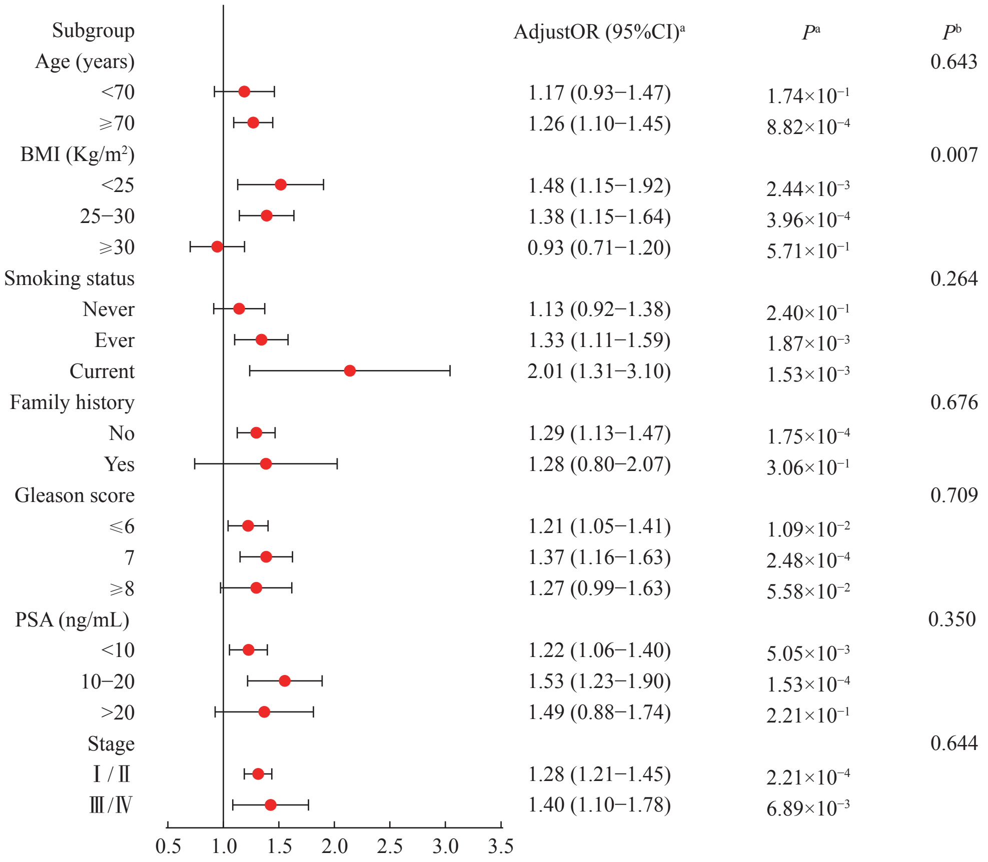

Supplementary Fig. 3 **Stratified analyses of demographic and clinicopathologic characteristics for the association between rs6001092 and prostate cancer risk in the dominant genetic model.** [a]*P*-values adjusted for age, body mass index, smoking status, and family history of prostate cancer in logistic regression model. [b]*P*-values for the heterogeneity. Abbreviations: OR, odds ratio; CI, confidence interval; BMI, body mass index; PSA, prostate specific antigen.

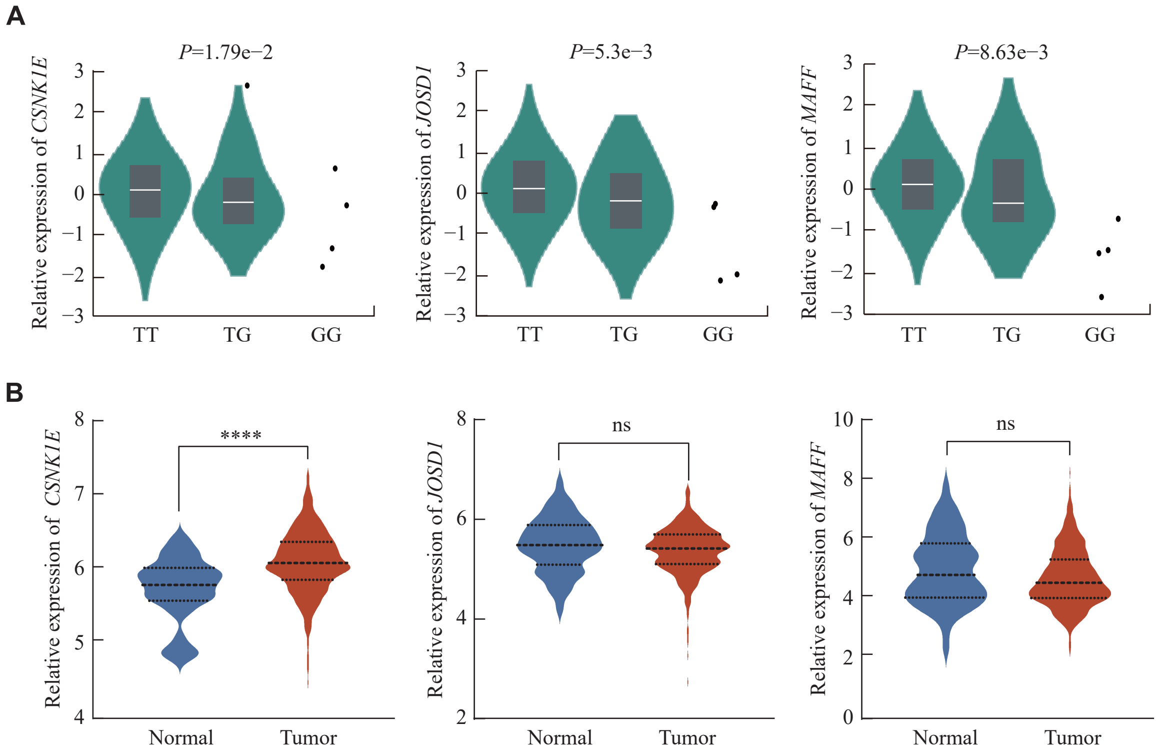

Supplementary Fig. 4 **The associations between rs6001092 and the expression levels of genes within a 1-Mbp window.** A: The expression of the significant genes in eQTL analyses between prostate tissues with TT, TG, and GG alleles in rs6001092 from the GTEx database. B: The expression of corresponding genes between tumor tissues and normal tissues in TCGA databases. The mRNA expression level of the genes was $\log_2(\text{TPM} + 1)$ transformed and presented as median and interquartile range. Two-tailed Student's *t*-test was used when normal distribution was met, and Mann-Whitney *U* test was used otherwise. $^{****}P < 0.000\,1$. Abbreviations: TCGA, The Cancer Genome Atlas; TPM, transcripts per million; ns, not significant.

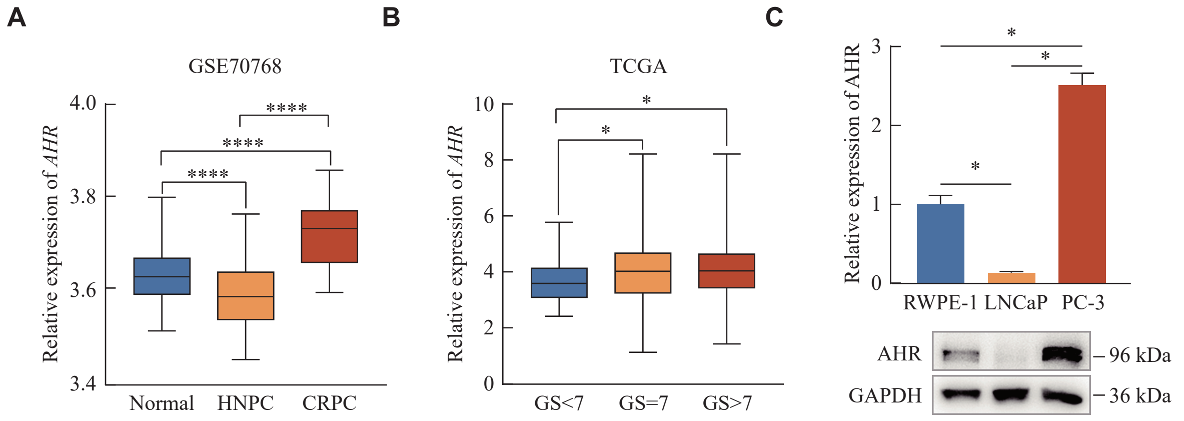

Supplementary Fig. 5 **The expression levels of *AHR* in prostate cancer tissues and cell lines.** A: The mRNA expression levels of *AHR* in hormone-naive prostate cancer (HNPC) tissues and castrate-resistant prostate cancer (CRPC) tissues in the GEO dataset GSE70768. B: The mRNA expression levels of *AHR* in patients with different Gleason scores in TCGA database. C: The mRNA and protein expression levels of AHR in prostate cancer and normal cell lines. Gene expression data were $\log_2$ transformed and presented as median and interquartile range. In prostate cancer cells, data are shown as the mean ± standard deviation values from three repeated experiments ($n = 3$). $^{*}P < 0.05$ and $^{***}P < 0.001$ by two-tailed Student's t-test. Abbreviation: GS, Gleason score.

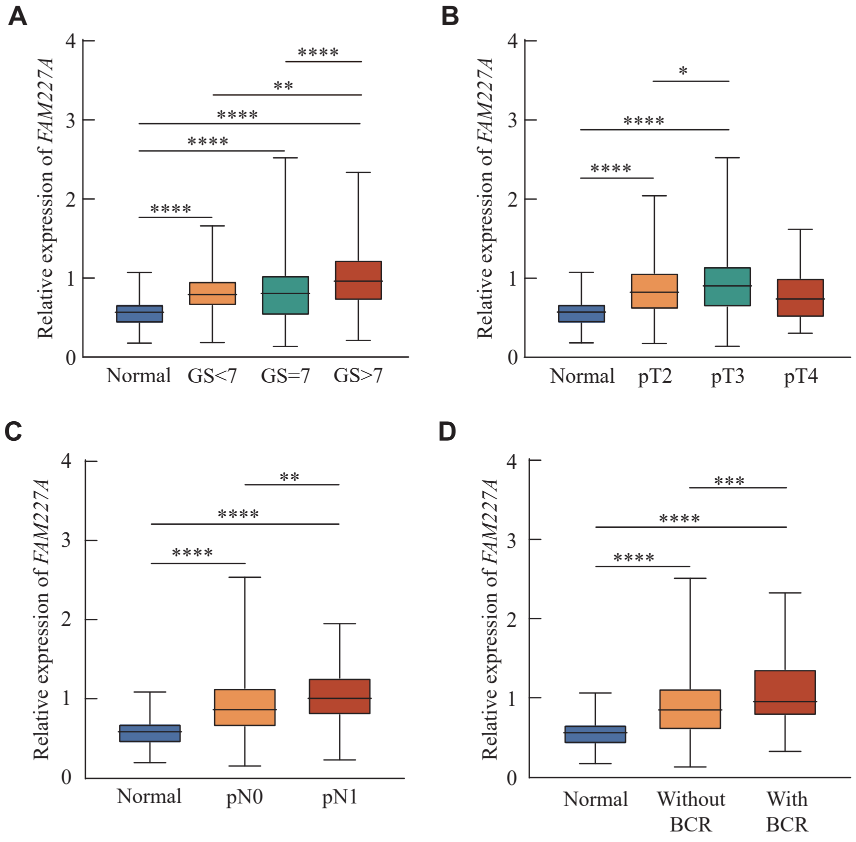

Supplementary Fig. 6 **The stratification analysis of *FAM227A* expression based on TCGA database.** A–D: Differences in *FAM227A* mRNA expression levels between normal and cancer tissues stratified by tumors' Gleason scores (GS), pathologic T stage (pT), pathologic N stage (pN), and the biochemical recurrence (BCR) status, respectively. The gene expression data were $\log_2(\text{TPM} + 1)$ transformed and presented as median and interquartile range. Two-tailed Student's t-test was used when normal distribution was met, and Mann-Whitney U test was used otherwise. $^{*}P < 0.05$, $^{**}P < 0.01$, and $^{***}P < 0.001$. Abbreviation: TPM, transcripts per million.

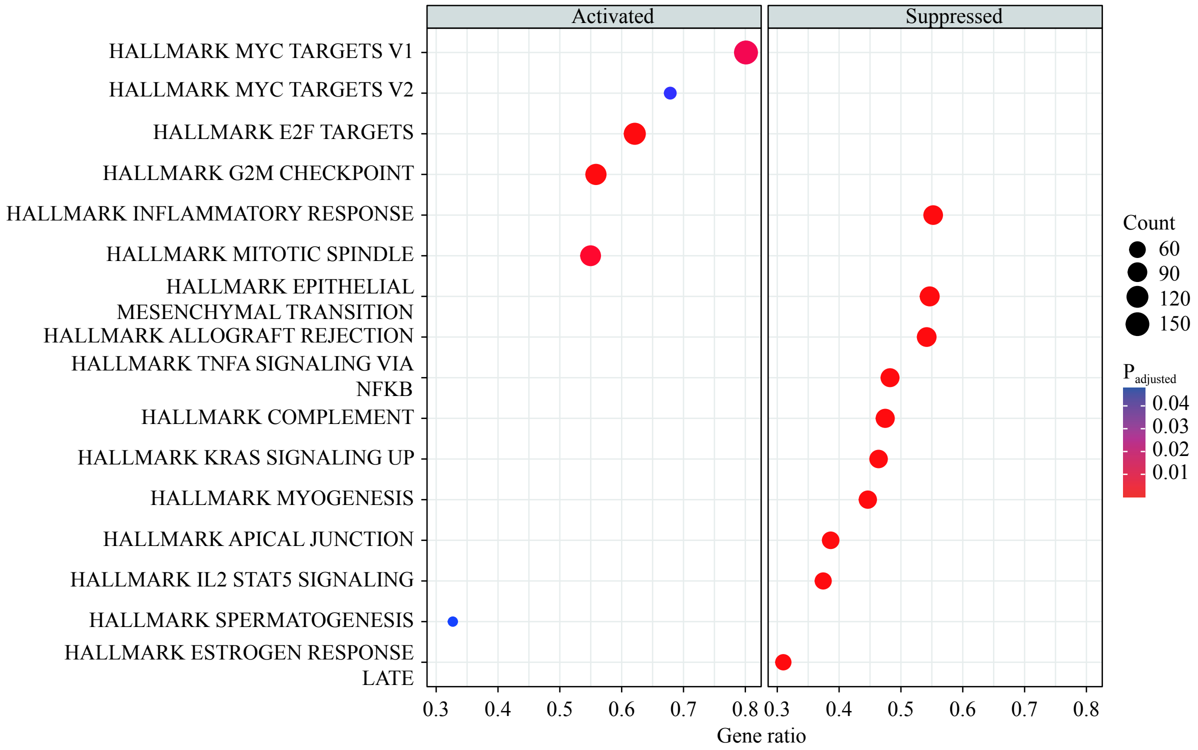

Supplementary Fig. 7 **Gene Set Enrichment Analysis (GSEA) for hallmark gene sets between high-expression and low-expression of *FAM227A*.** Significantly enriched activated and suppressed hallmarks terms.

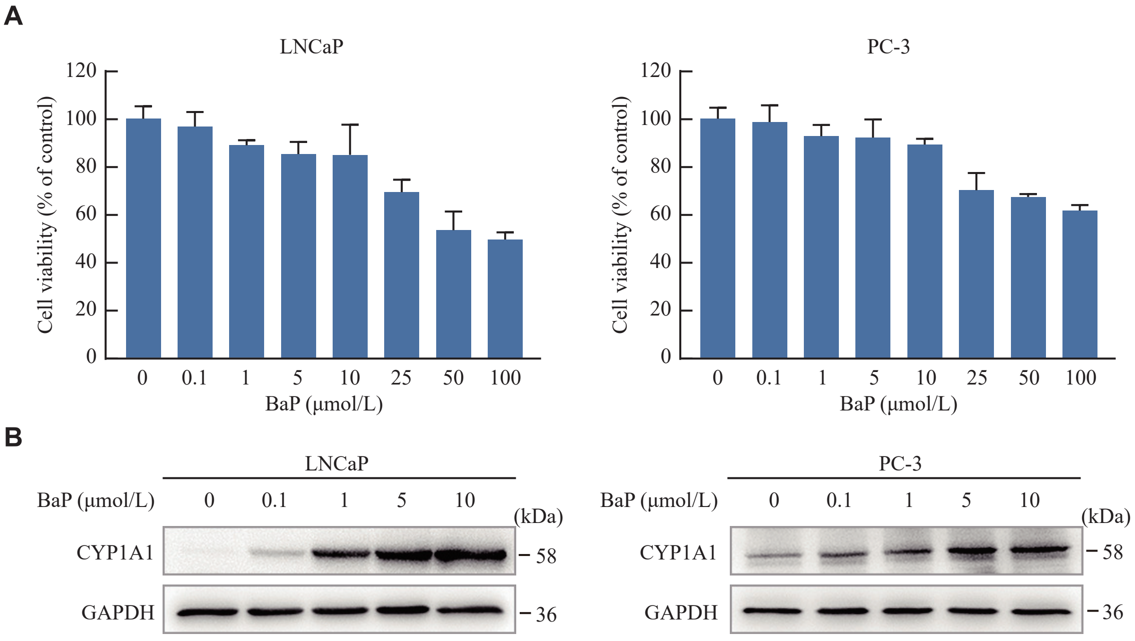

Supplementary Fig. 8 **Cell viability and AhR activation after treatment with different concentrations of BaP *in vitro*.** LNCaP and PC-3 cells were treated with the indicated concentrations of BaP for 24 h. A: Cell viability was examined by CCK-8 assay. B: The protein expression of CYP1A1 was detected by Western blotting. GAPDH was used as a loading control. Abbreviation: BaP, benzo[a]pyrene.

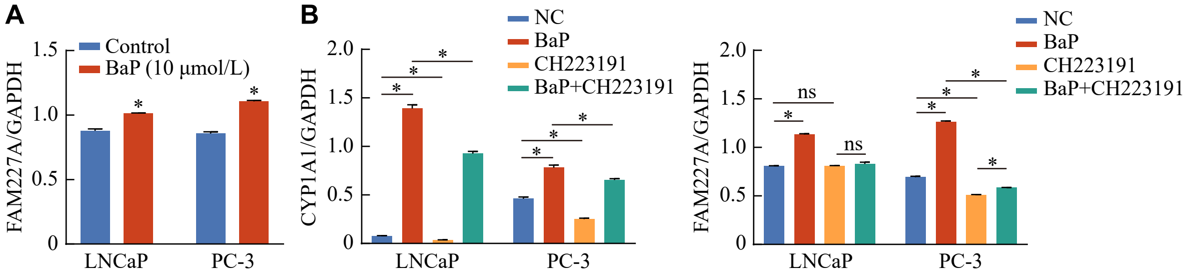

Supplementary Fig. 9 **Quantitative analysis of protein expression** A: Quantitative analysis of FAM227A protein expression. LNCaP and PC-3 cells were treated with BaP (10 μmol/L). B: Quantitative analysis of CYP1A1 and FAM227A protein expression. LNCaP and PC-3 cells were treated with BaP (10 μmol/L) and CH223191 (10 μmol/L) alone or in combination. Data are presented as mean ± standard deviation. $^*P < 0.05$ compared with the control group by two-tailed Student's *t*-test. ns, not significant. Abbreviation: BaP, benzo[a] pyrene.

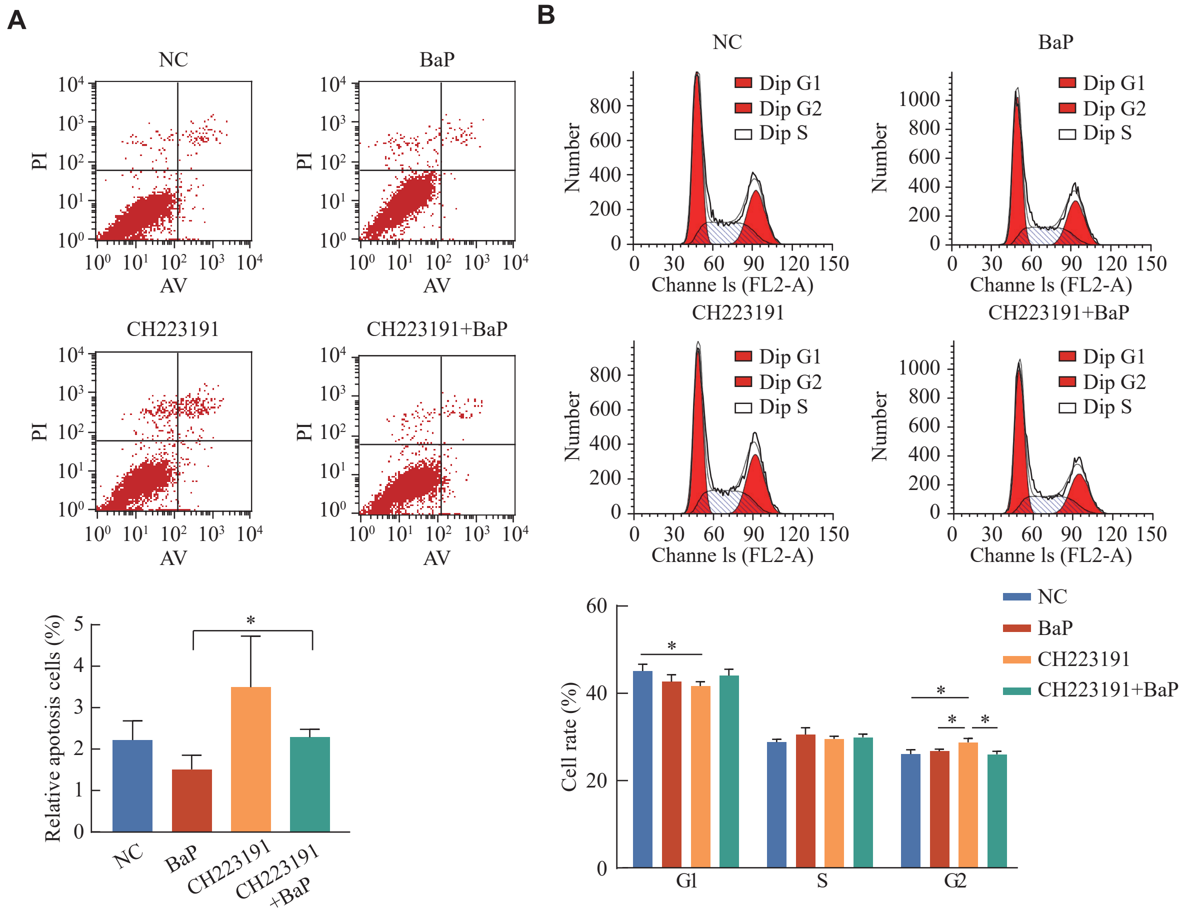

Supplementary Fig. 10 **Effects of BaP and inhibitor of AhR (CH223191) on prostate cancer cell apoptosis and the cell cycle distribution.** PC-3 cells treated with BaP (10 μmol/L), CH223191 (10 μmol/L), and BaP (10 μmol/L) combined with CH223191 (10 μmol/L), respectively. A: Flow cytometry detection of the apoptosis. B: Flow cytometry detection of the cell cycle. All of the experiments were performed in triplicate ($n = 3$). Data are presented as mean ± standard deviation. $^*P < 0.05$ compared with the control group by two-tailed Student's *t*-test. Abbreviation: BaP, benzo[a]pyrene.

Supplementary Table 1 **Baseline characteristics in prostate cancer cases and controls**

Variables	Cases (%) (*n*=4 662)	Controls (%) (*n*=3 114)	P^a
Age (years, mean±SD)[b]	68.74±5.80	75.30±5.36	$<1.00\times10^{-4}$
BMI (mean±SD)	27.33±3.79	27.65±4.09	4.27×10^{-4}
Smoking status			3.52×10^{-3}
Never	1 942 (41.66)	1 192 (38.28)	
Ever	2 355 (50.53)	1 634 (52.47)	
Current	364 (7.81)	288 (9.25)	
Missing	1	0	
Family History			$<1.00\times10^{-4}$
No	4 037 (88.53)	2 827 (93.09)	
Yes	523 (11.47)	210 (6.91)	
Missing	102	77	
Gleason Score			—
≤6	2 719 (59.02)		
7	1 453 (31.54)	—	
≥8	435 (9.44)		
Missing	55		
PSA (ng/mL)[c]			—
<10	3 630 (81.79)		
10–20	584 (13.16)	—	
>20	224 (5.05)		
Missing	224		
Stage[d]			—
Ⅰ/Ⅱ	4 066 (87.23)		
Ⅲ/Ⅳ	595 (12.77)	—	
Missing	1		

[a]*P* for two-side χ^2 test.
[b]Age at trial entry, computed from date of birth and randomization date.
[c]The PSA level from the most recent PSA test the participant received prior to diagnosis.
[d]Combined clinical and pathologic stage.
Abbreviations: SD, standard deviation; BMI, body mass index; PSA, prostate specific antigen.

Supplementary Table 2 **The sequences of the siRNAs**

siRNAs	Sequences (5′-3′)
si*FAM227A*-1	GCCAGAUCUUCUGUUAUAATT
si*FAM227A*-2	CCAGGGAAAUGAAGAAUAUTT
si*FAM227A*-3	GAGGGAAAGAGAAGAGAAATT
si*AHR*-1	GCUCUGAAUGGCUUUGUAUTT
si*AHR*-2	GCCACCAUCCAUACUUGAATT
si*AHR*-3	GCAGCUGAUAUGCUUUAUUTT

Supplementary Table 3 **The primer sequences for RT-qPCR**

Genes	Orientations	Sequences (5′-3′)
FAM227A	Forward	CCGGATAGCCCAGCACTATG
	Reverse	AGGTGCCTGAAATCCACAGG
AHR	Forward	ACATCACCTACGCCAGTCG
	Reverse	CGCTTGGAAGGATTTGACTTGA
CYP1A1	Forward	TCGGCCACGGAGTTTCTTC
	Reverse	GGTCAGCATGTGCCCAATCA
GAPDH	Forward	CCGGGAAACTGTGGCGTGATGG
	Reverse	AGGTGGAGGAGTGGGTGTCGCTGTT

Supplementary Table 4 **The probe sequences for EMSA**

Alleles	Orientations	Sequences (5′-3′)
rs6001092 T	Forward	TGCCATTGCACTCTAGCCTGGGT
	Reverse	ACCCAGGCTAGAGTGCAATGGCA
rs6001092 G	Forward	TGCCATTGCACGCTAGCCTGGGT
	Reverse	ACCCAGGCTAGCGTGCAATGGCA

Supplementary Table 5 **Associations between significant SNPs and prostate cancer risk**

SNPs	Chr	Positions	SE regions	Alleles[a]	MAF		$P_{(HWE)}$[b]	Adjusted ORs (95% CI)[c]	P^{c}	P^{d}
					Cases	Controls				
rs5750581	22	38695406	38679427–38724431	T/C	0.14	0.12	0.93	1.26 (1.12–1.41)	7.61×10^{-5}	3.90×10^{-2}
rs783390	6	106976289	106957879–106996592	G/A	0.34	0.33	0.57	1.14 (1.05–1.24)	1.27×10^{-3}	2.33×10^{-1}
rs13400438	2	118881001	118869123–118883582	G/A	0.40	0.42	0.10	0.88 (0.82–0.95)	1.36×10^{-3}	2.33×10^{-1}
rs4389803	6	2981772	2976198–2991690	A/G	0.45	0.43	0.06	1.12 (1.04–1.21)	3.40×10^{-3}	4.36×10^{-1}
rs627112	18	9609297	9594926–9684306	C/T	0.29	0.30	0.23	0.89 (0.82–0.97)	7.24×10^{-3}	6.24×10^{-1}
rs34791093	6	2985254	2976198–2991690	A/G	0.35	0.37	0.48	0.90 (0.83–0.97)	8.47×10^{-3}	6.24×10^{-1}
rs78022502	2	128396167	128371788–128397132	A/C	0.05	0.06	0.64	0.80 (0.67–0.94)	8.52×10^{-3}	6.24×10^{-1}
rs6776157	3	156543482	156531998–156546663	A/T	0.37	0.35	0.55	1.11 (1.03–1.21)	1.06×10^{-2}	6.80×10^{-1}
rs2171302	10	3815292	3781317–3830060	C/T	0.18	0.17	0.80	1.13 (1.02–1.26)	1.58×10^{-2}	9.01×10^{-1}
rs2000811	18	74809328	74800402–74844758	C/T	0.41	0.42	0.04	0.92 (0.85–0.99)	2.69×10^{-2}	9.13×10^{-1}
rs7204900	16	57645507	57644320–57680810	G/A	0.41	0.40	0.04	1.09 (1.01–1.18)	2.74×10^{-2}	9.13×10^{-1}
rs7313660	12	13054013	13022598–13057007	T/C	0.44	0.46	0.24	0.92 (0.85–0.99)	2.84×10^{-2}	9.13×10^{-1}
rs1417488	1	218523730	218512990–218610439	C/T	0.26	0.24	0.11	1.10 (1.01–1.21)	3.04×10^{-2}	9.13×10^{-1}
rs7943506	11	95987320	95919750–96021783	A/G	0.44	0.45	0.83	0.92 (0.85–0.99)	3.08×10^{-2}	9.13×10^{-1}
rs12533310	7	157082607	157064270–157111819	G/A	0.47	0.49	0.19	0.92 (0.85–0.99)	3.33×10^{-2}	9.13×10^{-1}
rs34627996	3	113276381	113270076–113296827	G/C	0.22	0.23	0.19	0.91 (0.83–0.99)	3.36×10^{-2}	9.13×10^{-1}
rs34354387	3	156506124	156482431–156514537	A/C	0.26	0.25	0.85	1.10 (1.01–1.20)	3.69×10^{-2}	9.13×10^{-1}
rs1110650	3	45653384	45634447–45669113	T/C	0.40	0.42	0.46	0.92 (0.85–1.00)	3.79×10^{-2}	9.13×10^{-1}
rs9887812	1	85744577	85744156–85836676	C/T	0.09	0.08	0.41	1.15 (1.01–1.31)	4.07×10^{-2}	9.13×10^{-1}
rs6023059	20	36737771	36737242–36816087	T/C	0.45	0.47	0.80	0.92 (0.86–1.00)	4.48×10^{-2}	9.13×10^{-1}
rs1864685	17	70721782	70672974–70723758	C/A	0.44	0.45	0.79	0.92 (0.85–1.00)	4.54×10^{-2}	9.13×10^{-1}
rs17185623	7	134050186	134048365–134054973	G/A	0.16	0.15	0.19	1.12 (1.00–1.24)	4.78×10^{-2}	9.13×10^{-1}
rs4456788	21	45616324	45592406–45638277	A/G	0.38	0.40	0.60	0.92 (0.85–1.00)	4.85×10^{-2}	9.13×10^{-1}
rs6791353	3	45685748	45681869–45718801	C/A	0.09	0.10	0.68	0.88 (0.77–1.00)	4.99×10^{-2}	9.13×10^{-1}

[a]Reference allele/effect allele.
[b]HWE Hardy Weinberg Equilibrium in control subjects.
[c]*P* for additive genetic model adjusted for age, body mass index, smoking status and family history of prostate cancer in logistic regression model.
[d]*P* after false discovery rate correction.
Abbreviations: SNPs, single nucleotide polymorphisms; MAF, minor allele frequency; HWE, Hardy-Weinberg equilibrium; SE, super-enhancer; OR, odds ratio; CI, confidence interval.

Supplementary Table 6 **Functional annotations of the SNPs in high LD with rs5750581 in *CSNK1E***

Chr	Positions	SNPs	Alleles[a]	r^2	RegulomeDB		HaploReg			3DSNP	
					Chromatin states	Motifs	Enhancer histone marks	DNAse	Motifs changed	Enhancers	Motifs
22	38695406	rs5750581	T/C	1	Strong transcription	0	11 tissues	8 tissues	EWSR1-FLI1	23	1
22	38695099	rs1534891	C/T	1	Strong transcription	2	13 tissues	9 tissues		22	0
22	38701094	rs6001092	T/G	0.95	Strong transcription	1	17 tissues	–	AhR	47	3
22	38703302	rs135755	C/T	0.85	Strong transcription	0	11 tissues	–	5 altered motifs	20	10

[a]Reference/effect allele.
Abbreviations: SNPs, single nucleotide polymorphisms; LD, linkage disequilibrium.

Supplementary Table 7 **Stratification analysis of demographic variables for the association between rs6001092 and prostate cancer risk in dominant genetic model**

Variables	Genotypes (Cases/Controls)		OR (95% CI)[a]	P^a	P^b
	TG/GG, *n* (%)	TT, *n* (%)			
Age[c]					0.643
<70	634/118 (25.15/22.61)	1 887/404 (74.85/77.39)	1.17 (0.93–1.47)	1.74×10^{-1}	
≥70	567/602 (27.62/23.78)	1 486/1 930 (72.38/76.22)	1.26 (1.10–1.45)	8.82×10^{-4}	
BMI (kg/m^2)					0.007
<25	326/163 (26.70/21.48)	895/596 (73.30/78.52)	1.48 (1.15–1.92)	2.44×10^{-3}	
25–30	631/352 (26.92/22.96)	1 713/1 181 (73.08/77.04)	1.38 (1.15–1.64)	3.96×10^{-4}	
≥30	227/198 (24.10/27.24)	715/529 (75.90/72.76)	0.93 (0.71–1.20)	5.71×10^{-1}	
Smoking status					0.264
Never	494/292 (25.92/24.77)	1 412/887 (74.08/75.23)	1.13 (0.92–1.38)	2.40×10^{-1}	
Ever	609/371 (26.41/23.29)	1 697/1 222 (73.59/76.71)	1.33 (1.11–1.59)	1.87×10^{-3}	
Current	98/57 (27.15/20.21)	263/225 (72.85/79.79)	2.01 (1.31–3.10)	1.53×10^{-3}	
Family history					0.676
No	1 054/656 (26.59/23.64)	2 910/2 119 (73.41/76.36)	1.29 (1.13–1.47)	1.75×10^{-4}	
Yes	121/40 (23.77/19.61)	388/164 (76.23/80.39)	1.28 (0.80–2.07)	3.06×10^{-1}	

[a]Adjusted for age, body mass index, smoking status and family history of prostate cancer in the dominant genetic model.
[b]*P*-value for heterogeneity.
[c]Age at trial entry, computed from date of birth and randomization date.
Abbreviations: OR, odds ratio; CI, confidence interval; BMI, body mass index.

Supplementary Table 8 **Stratification analysis of clinicopathologic variables for the association between rs6001092 and prostate cancer risk in dominant genetic model**

Variables	Genotypes		OR (95% CI)[a]	P^a	P^b
	TG/GG, *n* (%)	TT, *n* (%)			
Controls	720 (23.58)	2 334 (76.42)			
Cases	1 201 (26.26)	3 373 (73.74)	1.29 (1.13–1.50)	1.03×10^{-4}	
Gleason score					0.709
≤6	685 (25.67)	1 983 (74.33)	1.21 (1.05–1.41)	1.09×10^{-2}	
7	388 (27.21)	1038 (72.79)	1.37 (1.16–1.63)	2.48×10^{-4}	
≥8	111 (26.12)	314 (73.88)	1.27 (0.99–1.63)	5.58×10^{-2}	
PSA (ng/mL)					0.350
<10	920 (25.86)	2 637 (74.14)	1.22 (1.06–1.40)	5.05×10^{-3}	
10–20	168 (29.07)	410 (70.93)	1.53 (1.23–1.90)	1.53×10^{-4}	
>20	55 (25.23)	163 (74.77)	1.24 (0.88–1.74)	2.21×10^{-1}	
Stage					0.644
Ⅰ/Ⅱ	1 042 (26.13)	2 946 (73.87)	1.28 (1.21–1.45)	2.21×10^{-4}	
Ⅲ/Ⅳ	159 (27.18)	426 (72.82)	1.40 (1.10–1.78)	6.89×10^{-3}	

[a]Adjusted for age, body mass index, smoking status and family history in the dominant genetic model.
[b]*P*-value for heterogeneity.
Abbreviations: OR, odds ratio; CI, confidence interval; PSA, prostate specific antigen.

Supplementary Table 9 ***In silico*** **prediction of transcription factors with allele-specific binding to rs6001092 by JASPAR**

Regions	Matrix ID	Relative scores	Start	End	Strand	Predicted sequences
rs6001092	AHR	0.854	168	173	−	TGCTTG
The promoter region of *FAM227A*	AHR	0.918	965	970	−	GGCGTG
